# A Proposal for Comprehensive Audio-Vestibular Test Battery Protocol for Diagnosis and Follow-Up Monitoring in Patients with Vestibular Schwannoma Undergoing Surgical Tumor Removal

**DOI:** 10.3390/jcm13175007

**Published:** 2024-08-23

**Authors:** Patrycja Torchalla, Agnieszka Jasińska-Nowacka, Magdalena Lachowska, Kazimierz Niemczyk

**Affiliations:** Department of Otorhinolaryngology Head and Neck Surgery, Medical University of Warsaw, Banacha 1a Str., 02-097 Warsaw, Poland; patrycja.torchalla@wum.edu.pl (P.T.); mlachowska.wum@gmail.com (M.L.); kazimierz.niemczyk@wum.edu.pl (K.N.)

**Keywords:** cerebellopontine angle tumor, dizziness, posturography, vertigo, vestibular disorders

## Abstract

**Background:** A vestibular schwannoma (VS) is a benign tumor, causing audiological and vestibular symptoms. This study aimed to propose a comprehensive audio-vestibular test battery protocol for diagnosis and follow-up monitoring in patients with unilateral VSs undergoing surgical removal. **Methods:** The detailed interpretation of audiological and vestibular findings was presented in two example cases. The surgery was performed through the middle cranial fossa (#1) and translabyrinthine approach (#2). The participants were evaluated with tonal, speech, and impedance audiometry, ABR, caloric test, vHIT, cVEMP, oVEMP, SOT, and DHI. Patient and tumor characteristics were retrieved from the patient’s history. **Results:** In the postoperative period, the reduction in gain of the lateral semicircular canal was observed in the vHITs of both patients. The DHI in case #1 increased after surgery, while it decreased in case #2. The improvement in postural performances compared to the preoperative SOT (CON 5, CON 6, composite score) and immediately after the procedure was observed. **Conclusions:** A specific diagnostic protocol is necessary to compare the results of different surgical techniques and approaches. Diagnostic tests performed before the surgery should be repeated within a specific time frame during postoperative follow-up to enable the comparison of results. The proposed protocol can help us better understand the processes ongoing during tumor growth and postoperative vestibular compensation.

## 1. Introduction

### 1.1. Clinical Characteristics of Vestibular Schwannoma

A vestibular schwannoma (VS) is a benign tumor originating from Schwann cells surrounding the vestibular nerve, which slowly grows within the internal auditory canal and then into the cerebellopontine angle [1]. VSs account for approximately 6% of brain tumors and up to 80%–90% of cerebellopontine angle region tumors [2,3]. The clinical manifestations of VSs are varied. The most common symptoms presenting at diagnosis are unilateral sensorineural hearing loss (94% of patients) and tinnitus (83% of patients) [4,5,6,7,8].

Patients with VSs may present vestibular symptoms, including vertigo, dizziness, and sometimes postural instability, impacting patients’ quality of life [7,9]. The frequency of the vestibular symptoms varies widely, occurring in from 17% to 75% of patients, but they are likely underreported [5,10,11,12,13,14,15,16]. The slow, progressive alteration of vestibular function from the tumoral growth allows the gradual implementation of central adaptive mechanisms called vestibular compensation. As a result, patients rarely experience acute spinning vertigo episodes. They more commonly complain of mild to moderate chronic balance problems. However, some patients do not report any symptoms related to the balance system [17].

### 1.2. Surgical Treatment and Vestibular Compensation

VS surgery aims to achieve complete tumor removal with minimal postoperative morbidity and mortality. Depending on the tumor size and preoperative hearing level, the surgery can be performed through the middle fossa, translabyrinthine, or retrosigmoidal approach. VS surgery may lead to acute vestibular symptoms in the postoperative period due to the complete vestibular denervation causing decompensation of the previously compensated situation. Thus, most patients report severe vertigo immediately after surgery. Then, automatic progressive implementation of central adaptive mechanisms leads to vestibular and balance compensations, which occur within the first months after the denervation of the labyrinth. Interestingly, the course of the vestibular compensation varies individually. Some preoperative clinical factors, such as the patient’s age, physical activity, and tumor size, impact the balance compensation time [16,18,19,20]. The prevalence of persistent postsurgical disequilibrium ranges from 10% to 78% [1,21]. Nevertheless, further research is required to evaluate the role of these factors more accurately.

### 1.3. Aim of the Study

Various audiological and vestibular tests can be performed on patients before and after the surgery. A specific protocol should be established to assess the correlations between the clinical outcome and test results, including pre- and postoperative audio-vestibular tests. This study aimed to describe the comprehensive test battery protocol for diagnostics and postsurgical follow-up. The secondary aim was to present a detailed interpretation of audiological and vestibular findings in patients treated for a unilateral VS. For purposes of the test battery presentation and the detailed interpretation of test results, better understanding, and easier explanation, the protocol was presented in two example cases, one undergoing surgery through the middle cranial fossa approach and the other undergoing surgery through the translabyrinthine approach.

## 2. Materials and Methods

### 2.1. Ethical Consideration

The local Ethics Committee reviewed and approved the study protocol prior to the patients’ examination (AKBE/203/2022). The project conforms to The Code of Ethics of the World Medical Association (Declaration of Helsinki). This study includes a retrospective presentation of patients’ test results. No informed consent was required, and no patients’ personal information was divulged.

### 2.2. Patient Characteristics and Study Protocol

This study presented two patients as examples to illustrate the test battery protocol and detailed interpretation of audiological and vestibular outcomes. Both patients were diagnosed with a unilateral VS in the internal auditory canal and cerebellopontine angle, confirmed with gadolinium-enhanced magnetic resonance imaging (MRI) of the posterior cranial fossa, currently the gold-standard diagnostic method for VSs [7,22]. Preoperative MRI scans were used to measure tumor diameter in each patient. Tumor size was determined as the maximum tumor diameter based on which the patients were qualified as a specific Koos grade [23]. Both patients underwent ENT examination, including an otoneurological examination. Clinical symptoms and the results of the Dizziness Handicap Inventory (DHI) were evaluated during the preoperative diagnostics. The House–Brackmann score was used to assess facial nerve function before and after surgery ([24]—House JW, Brackmann DE. Facial nerve grading system. Otolaryngol Head Neck Surg 1985; 93; p. 146-7). Pure-tone audiometry, speech audiometry, impedance audiometry, auditory brainstem response (ABR), otoacoustic emission, videonystagmography (VNG) with the caloric test, the video head impulse test (vHIT), acoustic cervical and ocular vestibular myogenic potentials (cVEMP, oVEMP), and the computerized dynamic posturography with sensory organization test (SOT) were performed before surgical treatment.

Both patients underwent tumor resection performed by the same experienced otosurgeon. The middle cranial fossa approach was used in one of the patients (#1), while the translabyrinthine approach was performed in the other (#2). The histopathological examination confirmed the presence of VS.

**Figure 1:**
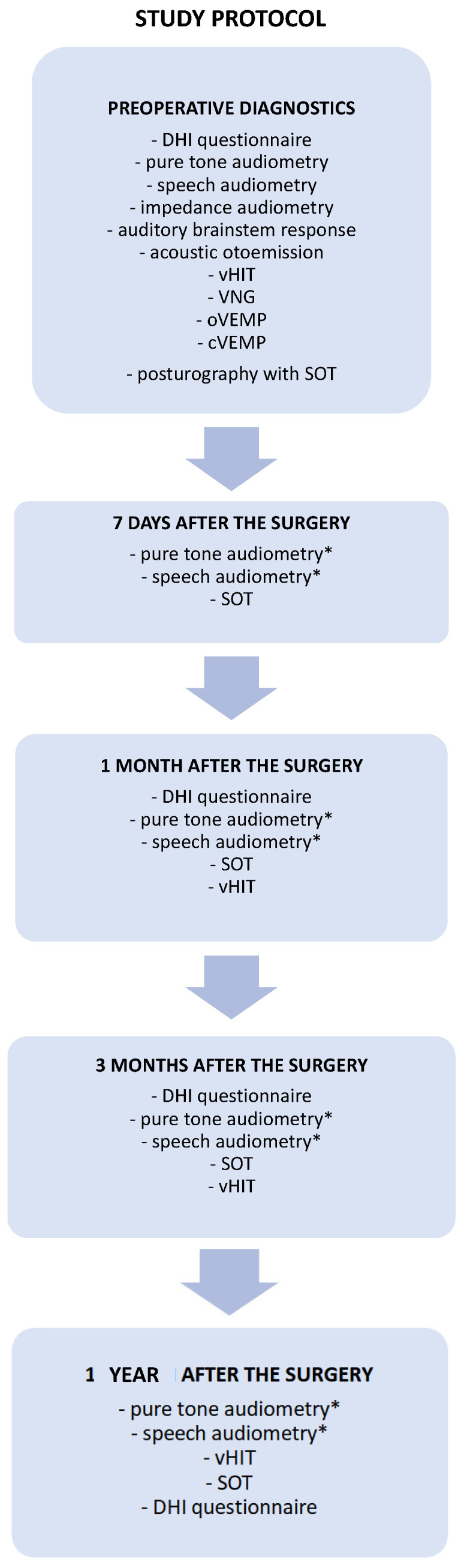
A proposal for comprehensive audio-vestibular test battery protocol for diagnosis and follow-up monitoring in patients with vestibular schwannomas undergoing surgical tumor removal. The set of diagnostics tests performed before and after the surgery necessary to monitor the audiological outcome and vestibular compensation. * Pure-tone and speech audiometry performed after the surgery only in patients treated through the middle cranial fossa approach. cVEMP—cervical vestibular myogenic potential, DHI—Dizziness Handicap Inventory, oVEMP—ocular vestibular myogenic potential, SOT—sensory organization test, vHIT—video head impulse test, VNG—videonystagmography.

The patients were controlled in the following time intervals: seven days, one month, three months, and one year after the tumor removal. The diagnostic and follow-up test battery protocol used in the present study is presented in detail in Figure 1. The audiological and vestibular tests were performed and analyzed by an experienced audiologist and an otolaryngologist.

### 2.3. Audiological Test Battery

Air-conduction hearing thresholds were measured for tonal stimuli at 125 to 8000 Hz frequencies in pure-tone audiometry. Bone-conduction hearing thresholds were measured at frequencies from 250 to 4000 Hz. The pure-tone average (PTA) hearing levels were calculated as the mean values among air-conduction hearing threshold levels at 500, 1000, 2000, and 3000 Hz. The AAO-HNS recommends this calculation method in assessing hearing status [25,26]. Moreover, the PTAs calculated according to 3 different methods (for 500, 1000, 2000, and 3000 Hz; for 500, 1000, 2000, and 4000 Hz; and for 500, 1000, and 2000 Hz) constitute the basis for coherent assessment of the hearing status in VS patients and may be used interchangeably in the determination of hearing capacity [27]. A monosyllabic word test was used in speech audiometry to assess speech detection, reception, discrimination thresholds, and word recognition scores at 65 dB SPL (normal speech conversational level).

Impedance audiometry was performed to evaluate middle ear function, and otoacoustic emission to assess cochlear function. In ABR, click stimuli at 90 dBnHL intensity and repetition rates of 11/s and 29/s were presented monaurally. Ipsilateral responses were collected and saved in 3 blocks of 1024 sweeps for each repetition rate. The results were then analyzed and classified into four groups: normal response, cochlear hearing loss pattern, retrocochlear hearing loss pattern, and absence of response.

### 2.4. Vestibular Test Battery

In videonystagmography (VNG) was performed using VisualEyes™ 525 system (Micromedical by Interacoustics), caloric responses were assessed after 30 °C and 44 °C water irrigation. Peak slow phase velocity (SPV) was used to evaluate the function of the lateral semicircular canals using Jongkees’ formula [28]. Results from the tumor side were evaluated compared to the contralateral healthy ear and divided into three groups: symmetrical caloric responses, unilateral caloric vestibular weakness defined as >25% asymmetry, and vestibular paresis on the affected side in case of bilateral peak SPV < 6°/s. Moreover, central vestibular dysfunction was assessed based on the oculomotor tests (gaze stability, saccades, smooth pursuit).

The video head impulse test (vHIT) examination was performed with VisualEyes™ EyeSeecam (Micromedical by Interacoustics) and included the standard protocol evaluating all six semicircular canals in three planes: the horizontal plane for lateral canals and the planes oriented along the right anterior, left posterior (RALP) canals and left anterior, right posterior (LARP) canals. In addition, a suppression head impulse paradigm (SHIMP) assessed the inhibition of the vestibulo-ocular reflex. The VOR was assessed using the ratio of eye velocity to head velocity (gain). Results from 0.8 to 1.2 were considered normal for the horizontal plane. Moreover, corrective saccades were evaluated on the affected and unaffected sides. Corrective saccades were divided into overt (occurring after the end of the head movement) and covert (occurring during the head movement). The organization of saccades was also analyzed—scattered and gathered patterns were distinguished [29,30].

In cervical vestibular evoked myogenic potentials (cVEMPs), the relaxation of the sternocleidomastoid muscle was provoked by acoustic stimuli of 500 Hz and 1000 Hz presented separately with an intensity of 95 dBnHL, triggering the saccule (inhibition). In ocular vestibular evoked myogenic potentials (oVEMPs), the activation of the inferior oblique muscle was evoked by the same acoustic stimuli one at a time, triggering the utricle. What is worth mentioning is that the oVEMPs are crossed neural pathways with the stimulus delivered to the left ear and the response recorded from the right eye and vice versa. The following VEMP parameters were assessed: P1 and N1 latency, peak-to-peak amplitude, and the amplitude asymmetry ratio between both measured sides. VEMPs were performed using Eclipse device (Micromedical by Interacoustics).

Computerized dynamic posturography was performed using NeuroCom device (EquiTest, NeuroCom) and included a sensory organization test (SOT), during which the patient’s task was to maintain an upright stance as stable as possible. The SOT protocol included six sensory tests arranged in different conditions from 1 to 6. The conditions’ descriptions are as follows: Condition 1—normal vision and fixed support, Condition 2—absent vision and fixed support, Condition 3—sway-referenced vision and fixed support, Condition 4—normal vision and sway-referenced support, Condition 5—absent vision and sway-referenced support, Condition 6—sway-referenced vision and sway-referenced support. Conditions 5 and 6 assess how patients use vestibular information when the only available sense provides reliable information. Reduced or distorted sensory information from the visual and somatosensory systems forces patients to rely on their vestibular sensations to maintain upright balance. Each condition was examined three times, and each trial lasted 20 s. The equilibrium score of each condition was the mean score of three consecutive trials. The following parameters were analyzed as a derivation of SOT sensory analysis scores calculated as presented in Table 1: the somatosensory ratio, the visual ratio, the vestibular ratio, the visual preference ratio, and the composite score (the weighted average of the scores of all conditions).

### 2.5. Dizziness Handicap Inventory

The assessment of quality of life was evaluated with the self-report using the Dizziness Handicap Inventory (DHI), a Polish-validated version [31]. The inventory consisted of 25 questions, which referred to the patient’s condition during the last month. Questions were designed to incorporate functional, physical, and emotional impacts on disability. According to the possible responses, there were scores to achieve for each question as follows: 0 points for an answer “no”, 2 for “sometimes”, and 4 for “always”. The maximum score was 100, and the minimum was 0 points. A higher score indicates a greater impairment in quality of life caused by vertigo or dizziness.

## 3. Results

### 3.1. Protocol Presentation in Example Cases with Detailed Test Results Interpretation

#### 3.1.1. Case Example—Patient #1

**Figure 2:**
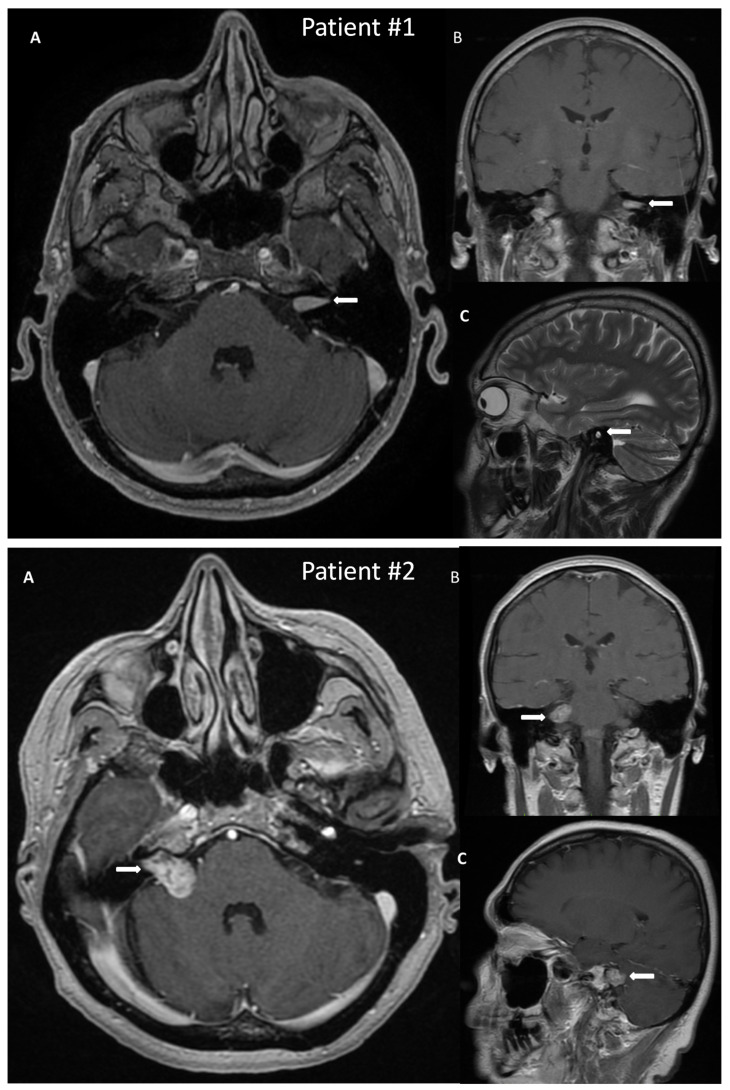
Post-gadolinium T1 magnetic resonance images of vestibular schwannoma in Patient #1, tumor located in the left internal auditory canal; and in Patient #2, tumor located in the right internal auditory canal protruding to the right cerebellopontine angle. The tumors are marked with arrows. (**A**)—axial, (**B**)—coronal, (**C**)—sagittal scans.

A 50-year-old male patient with a VS on the left side was admitted to the hospital for surgical treatment. On admission, the patient reported progressive hearing deterioration and a feeling of fullness in the left ear lasting many years. In addition, seven months before hospitalization, the patient experienced a single episode of vertigo accompanied by nausea and vomiting, which caused extensive diagnostic testing. The MRI of the posterior cranial fossa revealed a tumor in the left internal auditory canal of the size 14 × 6 mm, radiologically consistent with a schwannoma, Koos grade 1 (Figure 2). Preoperative facial nerve function bilaterally was assessed as I in the H-B scale. The patient did not suffer from any comorbidities.

Preoperative pure-tone audiometry revealed bilateral high-frequency sensorineural hearing loss; however, the PTA for the tumor side was 17.5 dB HL. The speech reception threshold was 45 dB SPL, and the speech recognition at 65 dB SPL was 100% for the tumor side. Impedance audiometry presented a type A tympanogram. ABR was abnormal for the tumor side, showing a retrocochlear hearing loss pattern.

**Figure 3:**
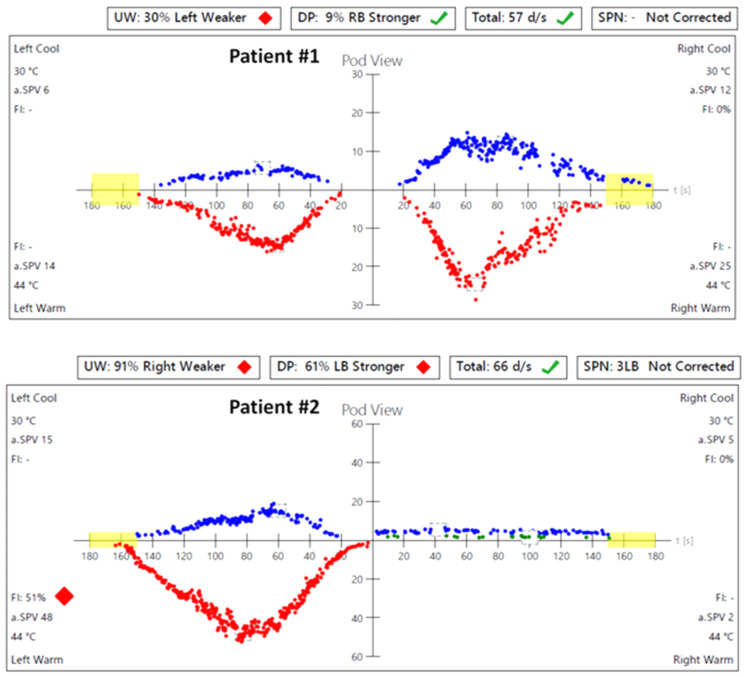
Videonystagmography (VNG)—caloric test results before the surgery: in Patient #1, a significant asymmetry of responses—weakness of the left labyrinth at 30%; in Patient #2, a significant asymmetry of responses—weakness of the right labyrinth at 91%. UW—unilateral weakness.

**Figure 4:**
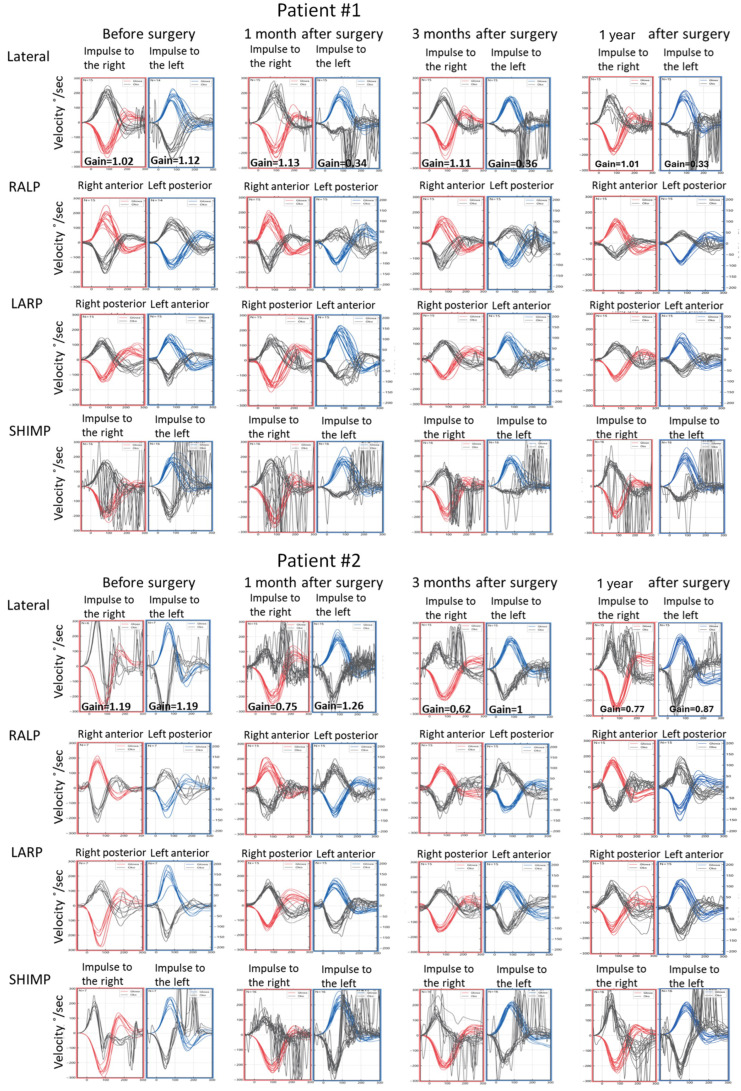
Video head impulse test (vHIT) results obtained before the surgical removal of the vestibular schwannoma, one month, three months, and one year after in Patients #1 (tumor on the left) and #2 (tumor on the right), respectively. Right color curves mark the movement of the head to the right side, blue color the movement of the head to the left side, and black color movement of the eyes. LARP—left anterior, right posterior canal; RALP—right anterior, left posterior canal; SHIMP—suppression head impulse test.

**Figure 5:**
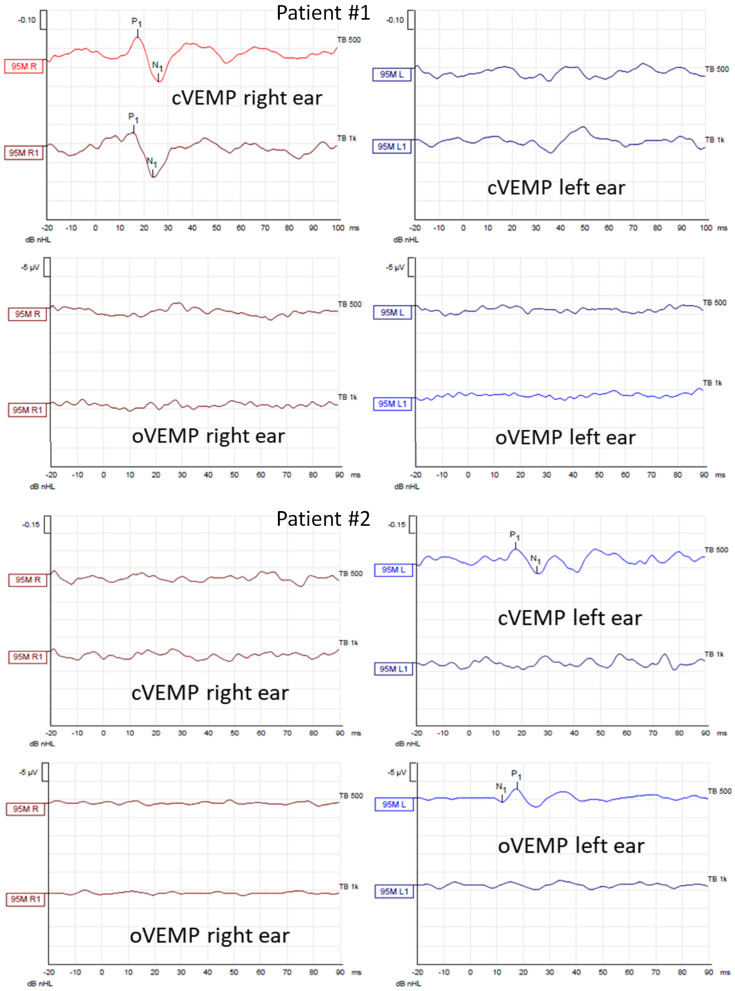
Air–conducted cervical and ocular vestibular evoked myogenic potentials (cVEMP and oVEMP) recordings obtained from Patients #1 (tumor on the left) and #2 (tumor on the right) before surgical vestibular schwannoma removal. In each patient, the first row shows cVEMP and the second oVEMP. The first column shows responses from the right ear and the second from the left ear. In each recording, the waves P1 and N1 are marked if present. cVEMPs and oVEMPs were recorded using stimuli of 500 Hz and 1000 Hz with an intensity of 95 dBnHL.

**Figure 6:**
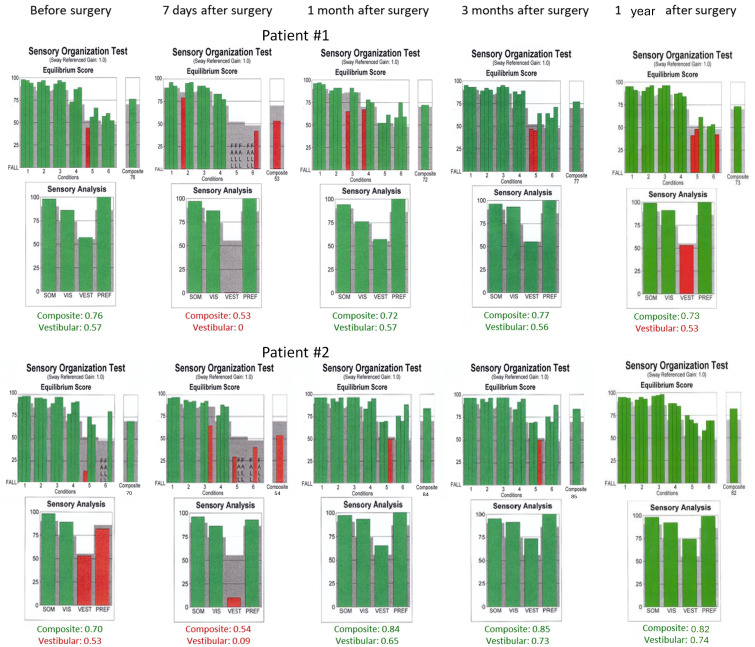
Sensory organization test (SOT) results obtained before the surgical removal of the vestibular schwannoma one month, three months, and one year after in Patients #1 (tumor on the left) and #2 (tumor on the right), respectively. Results within normal range are green. SOM—somatosensory, VIS—vision, VEST—vestibular, PREF—visual preference.

In caloric tests, a significant asymmetry of responses was detected due to the weakness of the left labyrinth at 30% (Figure 3). The vHIT showed overt corrective saccades of small amplitude despite a gain value within the normal range while examining the lateral plane with the head moving to the right. The gain was 1.12 for the left side (tumor). Testing RALP and LARP planes showed symmetrical and normal results with the absence of the corrective saccades for each semicircular canal (Figure 4). Before surgery, the patient underwent cVEMP and oVEMP tests. cVEMP testing showed responses only for the unaffected ear for the 500 Hz and 1000 Hz stimuli. No reproducible responses were observed in the oVEMP test for the 500 Hz and 1000 Hz stimuli bilaterally. (Figure 5). The result of the posturography was normal, with normal scores in every condition (Figure 6). The patient scored 0 points in the DHI.

The patient was advised for surgical removal of the left vestibular schwannoma through the middle cranial fossa approach. The transtympanic electrocochleography was performed during the surgery to monitor the hearing. During the surgery, the tumor was localized as arising from the inferior vestibular nerve and was removed in one piece. Immediately after the surgery, the facial nerve paresis on the left side was assessed as grade III using the H-B score. Histopathological examination confirmed that the tumor originated from Schwann cells.

No hearing deterioration was found in postoperative pure-tone and speech audiometry tests performed on the 7th day after the surgery. Due to intraoperative hearing monitoring through the tympanic membrane, the hemotympanum and tympanic perforation were found. Hence, the air-bone gap around 25 dB was observed in pure-tone audiometry. On the 8th day, the patient was discharged home in good general condition, with a recommendation to continue the rehabilitation of facial mimic muscles on the left side.

During follow-up visits one month and three months after the surgery, the patient reported periodic balance disorders with subjective deviation to the left side while walking. Symptoms intensified with rapid changes in body position, particularly the head, and excessive physical exertion. One year after surgery, the patient did not report any symptoms. The facial nerve function on the left side was assessed as grade II on the H-B scale. The PTAs for the tumor side were 22.5 dB HL, 22.5 dB HL, and 26.25 dB HL one month, three months, and one year after surgery, respectively.

Postoperative vHIT examinations showed the presence of corrective covert and overt saccades presenting a gathered pattern and reduction in the gain value to 0.34 (one month after surgery), 0.36 (three months after surgery), and 0.33 (one year after surgery) when examining the semicircular canals with the head tilted to the left, which is a result typical of patients with damage or denervation of the labyrinth. Also, corrective overt saccades were observed in examining the left posterior semicircular canal (Figure 4). The result of the SOT testing initially deteriorated on the 7th day after the tumor removal, showing abnormal results in Conditions 5 and 6. However, after one and three months, the SOT results showed the general balance returned to normal, with normal Conditions 5 and 6, vestibular, and composite scores. One year after surgery, a deterioration of results in Condition 5, Condition 6, and the vestibular score was noticed; however, the composite score remained normal (Figure 6). The postoperative DHI was 6 points one and three months after surgery and 0 points one year after surgery. The patient remained under the care of the ENT outpatient department.

#### 3.1.2. Case Example—Patient #2

A 54-year-old female with a VS on the right side was admitted for surgical treatment. On admission, the patient reported progressive hearing loss with a periodic feeling of fullness, congestion in the right ear, and fluctuating balance disorders in the form of ground instability. In addition, she reported incidental pain in the right temporal region. The onset of symptoms was about 18 months before hospitalization. The MRI with contrast revealed a tumor of 20 × 14 mm size, with features of a VS, located in the right cerebellopontine angle and the right internal auditory canal, Koos grade 3 (Figure 2). As for comorbidities, the patient suffered from hypercholesterolemia.

Preoperative pure-tone audiometry revealed sensorineural hearing loss on the tumor side with a PTA of 93.75 dB HL. The speech detection threshold was not achieved, and the word recognition score at 65 dB was 0%. The impedance audiometry presented a type A tympanogram. The complete absence of response on the right side was observed in ABR.

In caloric tests, a significant asymmetry of responses presented weakness of the right labyrinth at 91% (Figure 3). The preoperative vHIT revealed the covert and overt saccades in the right lateral semicircular canal with a normal gain of 1.19 (Figure 4). Results of the cVEMP testing showed normal response for the left ear and no response for the right for the 500 Hz stimulus. No response bilaterally was observed for the 1000 Hz stimulus. oVEMP testing revealed a normal response for the left ear and no response for the right for the 500 Hz stimulus. No response was observed for the 1000 Hz stimulus bilaterally. (Figure 5). Before surgery, the SOT revealed a vestibular pattern of abnormal results, with the composite score within the normal range (Figure 6). The patient scored 12 points in the DHI.

The patient underwent surgical removal of the tumor with a translabyrinthine approach. Intraoperatively, the inferior vestibular nerve origin of the tumor was confirmed. In the histopathological examination, a VS was confirmed. The surgery was complicated by paresis of the facial nerve on the right side, confirmed postoperatively as degree V using the H-B scale.

In SOTs performed seven days after surgery, there were decreased equilibrium scores for vestibular function presented in Conditions 5 and 6, vestibular and composite scores (Figure 6). On the 9th day, the patient was discharged home in a stable general and local condition, with a recommendation to continue the rehabilitation of the facial mimic muscles.

During follow-up visits one month, three months, and one year after the surgery, the patient reported periodic balance disorders with a deviation to the right, which intensified during rapid changes in body position and after intensive physical effort. In addition, the patient reported regression of the feeling of fullness and congestion of the right ear (previously the tumor side). The facial nerve function on the right side was graded IV on the H-B scale one and three months after surgery, while one year after surgery, this improved to grade III.

As for the right lateral semicircular canal, one month after surgery, the vHIT revealed scattered covert and overt saccades with a gain of 0.75, and only covert saccades with gains of 0.62 and 0.77 were revealed three months and one year after surgery, respectively (Figure 4). During follow-up visits one month, three months, and one year after the surgery, an improvement in postural performances compared to the preoperative SOT and immediately after the procedure was observed (Figure 6). The composite and vestibular scores and results in Conditions 5 and 6 were normalized. The DHI was 6 points after one month and 8 points after three months and after one year.

## 4. Discussion

Balance and hearing deterioration significantly impact the quality of life in patients with a unilateral VS. Vertigo and dizziness seem more damaging than hearing symptoms in this group of patients [32]. However, the complex nature of the anatomy and physiology of the vestibular system necessitates the need for multiple examinations. A protocol that sets diagnostic and follow-up tests in the proper order and time sequence to be performed in this group of patients seems essential to better understand the impact of a tumor growing on the vestibular nerve on the balance system and balance compensation after its removal.

Although the vestibular tests do not present a high sensitivity for detecting VSs, and results might be nonspecific, they are of great interest to many researchers as they are helpful in the functional evaluation of the balance system. It was already shown in the literature that VS induce hearing loss independently of the tumor size [26], but the deterioration of vestibular function increases with tumor size [10]. Tumor size correlates with impairment of the vestibulo-ocular reflex in the vHIT [33]. Moreover, abnormal VNG, vHIT, or VEMP results should alert physicians to consider MRI in patients presenting nonspecific disequilibrium or vertigo [34]. Moreover, vestibular tests are very helpful in the presurgical evaluation of the vestibular system and postoperative follow-up.

Tumors of the vestibular nerve can cause the gradual implementation of central adaptive mechanisms called vestibular compensation, which minimizes vestibular symptoms during slow tumor growth. However, some patients may suffer from acute vertigo, while others report intermittent imbalance problems or do not have any symptoms. There is no specific neurotological clinical representation of patients with cerebellopontine angle tumors. Vestibular manifestations in patients with VSs might be variable. VSs present with a progression of vestibular dysfunction, which can generally be asymptomatic because of the timely establishment of vestibular compensation [4]. A single vestibular function test result often contradicts a patient’s clinical symptoms. Thus, a set of diagnostic neurotological examinations is required. In the literature, authors presented patients with no typical symptoms of vertigo but still a significant decrease in the gain in vHIT results [35] and unilateral weakness in the caloric test at the affected semicircular canal [36]. In this study, we aimed to present the audio-vestibular test battery protocol and interpret in detail the outcomes before and in short-term follow-up times after VS surgery to add to a better understanding of the auditory vestibular characteristics of a VS. Moreover, our study’s objective was to propose a protocol for a set of tests that might be helpful in VS diagnostics and postoperative follow-up balance assessment. The constant development and improvement of vestibular diagnostic tests effectively support the evaluation of peripheral vestibular disorders [37].

The caloric test is the most popular method to evaluate the vestibular system. However, it assesses vestibular function at ultralow frequencies (0.025 Hz) not used in daily activities. Moreover, the water and air used in this method are not physiological stimuli. The caloric test unilaterally stimulates the lateral semicircular canal function, which the superior vestibular nerve innervates [38]. There may be a significant unilateral weakness in caloric response when a VS originates from the superior branch of the vestibular nerve. Borgmann et al. [39] tested 111 patients with a VS preoperatively. They defined pathologic caloric response as an indicator of the involvement of superior vestibular nerve schwannomas and the normal finding as a sign of inferior vestibular schwannomas. Their study suggested that caloric tests can help predict nerve origin in VS patients and could indirectly predict hearing preservation because patients with superior vestibular nerve tumors have less postoperative hearing loss than patients with inferior vestibular nerve tumors.

Another test is the HIT, which can be easily performed during the bedside examination with its modification. The vHIT evaluates high frequencies (2–6 Hz) more commonly during normal head movements. The vHIT is used to detect lesions of all semicircular canals, and the test’s sensitivity to VSs is 80%, which suggests that the vHIT could be used as a screening tool for VSs, owing to its convenience [34]. It is an easy tool to examine vestibulopathy and should be performed in every case, as suggested in the literature [8,30]. The vHIT can effectively evaluate the origin of a VS through the VOR, detecting the severity of function loss in the affected semicircular canal and monitoring the progression of the VS [17].

VEMPs may also be helpful in the evaluation of both superior and inferior branches of the vestibular nerve. To evaluate the saccule and inferior vestibular nerve function, the cVEMP is used, and the oVEMP is used to reflect the function of the utricle and superior vestibular nerve. Thus, they may help identify the VS’s superior/inferior vestibular origin and monitor tumor progression, as shown in the literature [7,40,41,42,43].

Taylor et al. [34] reported that patients with schwannomas sized >14 mm had at least two abnormal vestibular test results among the three performed (i.e., cVEMP, oVEMP, and vHIT), e.g., He et al. [44] showed no response to the caloric and VEMP tests on the affected sides in patients with a large unilateral VS [34].

In our study, Patient #1, with a VS on the left originating from the inferior vestibular nerve, presented 30% unilateral weakness in the caloric test on the affected side. Moderate asymmetry may result from mild tumor compression on the superior nerve with partial lateral semicircular canal function preservation. The VNG result was consistent with the vHIT of the lateral plane, where a normal gain value was shown. However, some corrective saccades of small amplitude were present during head movement to the right. Analyzing their morphology, latency, and amplitude, considering the lack of saccades present during head movement toward the side of the tumor, they are not typical for vestibular patterns of abnormal vHIT results. Surprisingly, the results of anterior and posterior semicircular canals were normal. Still, the patient presented no responses in oVEMP and cVEMP on the affected side. A normal vHIT result of the posterior semicircular canal is inconsistent with abnormal cVEMP on the same side as both tests evaluate structures innervated by the inferior branch of the vestibular nerve. However, different types of stimuli and different receptors are examined with varied frequency ranges in vHITs and VEMPs. Presumably, e.g., slowly growing small VSs may affect only some of the fibers of the inferior vestibular nerve, which may result in only partial loss of function. However, this hypothesis requires further studies with a larger group of patients.

Patient #2 had a VS originating from the inferior vestibular nerve branch on the right side. She had abnormal oVEMP, cVEMP, and 91% unilateral weakness in the caloric test on the affected side. In the vHIT, during the head movement toward the side of the tumor, saccades in the lateral semicircular canal were visible. The SHIMP protocol showed an asymmetry with less normal saccades on the right side. According to the Koos scale, the tumor was larger and more advanced in this patient than in the previous patient. Due to the tumor’s progression, the superior vestibular nerve may be more compressed in time, resulting in the loss of function. Analyzing the vHIT of the RALP and LARP planes, single corrective saccades in both posterior semicircular canals were visualized. Because of their symmetrical character and lack of repeatability, they might not indicate a loss of function of the posterior semicircular canals and the inferior vestibular nerve, and they may be only artifacts.

Analyzing postoperative results of the vHIT, multiple corrective saccades were present in all semicircular canal results on the affected side, which is typical for acute vestibular denervation. Multiple covert saccades and a clustered pattern in the subsequent examinations might indicate progressive vestibular compensation.

The balance can be evaluated comprehensively using dynamic posturography, which does not assess the responses of the vestibular organ but still provides information about somatosensory, visual, and vestibular function in maintaining overall body balance [45]. Posturography is well known, and the SOT provides crucial information about a patient’s ability to maintain a stable posture. The SOT identifies balance deficits and distinguishes which system (somatosensory, visual, or vestibular) is disturbed in a patient with a VS [46]. With six conditions, the SOT assesses sensory integration in sensory conflict situations. Patients with VSs who presented vertigo, dizziness, or imbalance symptoms scored lower in CDP testing than those without vestibular symptoms [47]. Patients with VS scored lower in the SOT than healthy patients [20]. Gouveris et al. [47] presented significant differences in the distribution of Conditions 5 and 6, which evaluate vestibular function of VS patients with and without subjective vestibular symptoms. In our study, Patient #2 with symptoms tended to have lower scores in Conditions 5 and 6 than Patient #1 without balance symptoms, although this trend was insufficient for reliable discrimination for all patients. Conditions 5 and 6 assess how patients use vestibular information when the only available sense provides reliable information. Reduced or distorted sensory information from the visual and somatosensory systems forces patients to rely on their vestibular sensations to maintain upright balance.

In the present study, Patient #1 presented normal SOT results before surgery, consistent with his DHI score, which was 0, and the vestibular test results. One week after VS removal, the vestibular pattern was prominent, with abnormal Conditions 5 and 6 and composite score. After one month and three months after surgery, the patient’s SOT returned normal, indicating vestibular compensation when analyzed with the DHI questionnaire (6 points) and postoperative vHIT results with gathered saccades. One year after surgery, the overall body balance was normal, with an abnormal vestibular score; however, the DHI score was 0 points, indicating no subjective vertigo or dizziness. Patient #2’s vestibular pattern presented abnormal results before the surgery despite normal composite results in the SOT. Compared to the previous patient, in this patient, greater tumor advancement was associated with more severe symptoms (12-point score on the DHI questionnaire) and asymmetrical vestibular results. The SOT results deteriorated in an early (one week) postoperative period, showing abnormal results with Conditions 5 and 6 as typical findings for vestibular dysfunction. This vestibular dysfunction was resolved after one month, three months, and one year of surgery, consistent with a lower DHI score and covert saccades in the vHIT. Thus, the SOT can be used during follow-up visits to quickly assess the vestibular compensation in patients after VS surgery.

Moreover, the SOT results are presented in a graphical format that can be shown to the patients and is quite easy to understand. Thus, improving the SOT results may positively impact the psychological aspect of patients’ recovery and facilitate their return to daily activities. In the present study, the SOT was repeated on every follow-up visit as the results can be reliably compared to the previous tests and do not cause patient discomfort in the early postoperative period. The vHIT examination was performed in preoperative diagnostics and during follow-up visits one month, three months, and one year after the surgery. It was decided not to perform the vHIT one week after the surgery as it could cause discomfort in the operated area due to the tight band of the goggles. It is necessary to perform follow-up tests within a specific time frame to compare them to the preoperative results. Nevertheless, the patient’s comfort should be considered when establishing a diagnostic protocol, as it may affect compliance.

With VS removal through the middle cranial fossa and a retrosigmoid approach, it is possible to achieve long-term hearing preservation of up to 80% [48,49,50,51,52,53,54]. Hearing outcomes after VS treatment are well described in the literature. However, the vestibular function and functional level of patients after surgical treatment of VSs are still not fully understood.

Parietti-Winkler et al. [19,55,56] have shown that the preoperative vestibular status could modify the postsurgery postural compensation. Patients with poor vestibular function before surgery recover earlier than those with normal vestibular function [57]. A high vestibular asymmetry before surgery can cause the implementation of central vestibular compensatory mechanisms, which develop with tumor growth even before surgery. These compensatory mechanisms were modified by surgery-related decompensation after tumor removal, but the neural networks remained present and could serve as a neuroanatomical support to balance compensation.

In contrast, the normoreflexy status did not lead to the preoperative implementation of new neural networks because of insufficient asymmetry to induce the compensation-related mechanisms. In this respect, unilateral vestibular differentiation induced a sudden and huge asymmetry, leading to high balance disturbances. Our study described two examples of patients with episodes of vertigo and imbalance disorders before surgery despite differences in the tumor size and preoperative caloric test results. In the context of complex innervation of the labyrinth, caloric tests are insufficient to fully evaluate the vestibular system in patients with vestibular nerve tumors.

Moreover, some studies showed an association between physical activity before the surgery and postoperative short-term balance performances. The literature confirms that preoperative physical activity can promote the neuroplasticity of neural networks involved in motor learning. Therefore, preoperative physical activity could enhance the postoperative quality of life and speed up the recovery of postural performance, but also reduce medical management and societal costs, with a faster and easier return to daily life activities and work [55].

The protocol described in this study consists of preoperative and postoperative audiological and vestibular tests, allowing for monitoring the postoperative audiological status and vestibular compensation in patients after the surgical removal of vestibular schwannomas. Repeated follow-up examinations may be helpful for the patients to be aware of their progress in compensation as they give evidence of improvement in balance and dynamic visual acuity and encourage the patients to engage in daily activities. That is crucial because the psychological aspect is important in functional improvement in patients with vestibular disorders [58].

## 5. Conclusions

Modern vestibular function tests can determine the laterality, affected frequencies, and lesion nerve of origin, providing a basis for VS screening, diagnosis, and postsurgical follow-up. In this study, the example cases showed that surgical treatment through the middle cranial fossa and the translabyrinthine approach enables overall balance recovery. However, only the middle cranial fossa approach allows for hearing preservation. A set of diagnostics tests performed before and after the surgery is necessary to monitor the audiological outcome and vestibular compensation in patients after surgical removal of VSs. The specific diagnostic protocol is necessary to compare the results of different surgical techniques and approaches.

Moreover, diagnostic tests performed before the surgery should be repeated within a specific time frame during postoperative follow-up to enable the comparison of their results. Multiple diagnostic tests examining individual components of the vestibular system contribute to a better understanding of processes occurring during tumor growth and postoperative vestibular compensation. However, further research on a larger group of patients is required to allow the comparison of vestibular compensation depending on the clinical course and surgical technique.

## Figures and Tables

**Table 1:** The derivation of the SOT sensory analysis scores. SOT—sensory organization test.

Sensory Analysis Scores	Ratio of Equilibrium Scores
Somatosensory (SOM)	Condition 2/Condition 1
Vision (VIS)	Condition 4/Condition 1
Vestibular (VEST)	Condition 5/Condition 1
Visual Preference (PREF)	Conditions 3 + 6/Conditions 2 + 5
Composite score (COM)	weighted average of the scores of all conditions

## Data Availability

Dataset available on request from the authors.
